# Surveillance of West Nile virus infections in humans and animals in Europe, monthly report – data submitted up to 5 August 2026

**DOI:** 10.2903/j.efsa.2026.10304

**Published:** 2026-08-18

**Authors:** European Centre for Disease Prevention and Control (ECDC), European Food Safety Authority (EFSA)

**Keywords:** birds, equids, humans, outbreak, West Nile virus

## Abstract

**Findings from human surveillance:**

In 2026, and as at 5 August, seven countries in Europe have reported 245 locally acquired^1^ human cases of West Nile Virus (WNV) infection. The earliest and latest date of onset were on 12 May 2026 and 1 August 2026, respectively. Locally acquired cases have been reported by **Italy** (139 cases), **Greece** (65, of which four with unknown place of infection), **Spain** (17 cases), **North Macedonia** (13 cases), **Romania** (six cases), **France** (four cases) and **Germany** (one case). In Europe, 12 deaths have been reported by **Greece** (six deaths), **Italy** (five deaths) and **Romania** (one death).

The number of human cases reported so far (245 cases) is below the average for the corresponding period over the past decade (403 cases). However, reporting delays may result in an under‐estimation of the current burden, as the 2026 data remain provisional, whereas the ten‐year average is based on consolidated data from previous years. In addition, this ten‐year average is influenced by several particularly intense WNV transmission seasons, notably in 2018 (1065 cases reported up to the corresponding week), 2022 (781 cases) and 2024 (654 cases). To date, most cases have been reported in **Italy** (139 cases) and **Greece** (65 cases). Although the number of cases reported in Italy is lower than during the same period in 2025 (168 cases), it remains the most affected country in Europe in 2026. **Greece** has reported more cases in 2026 than during the same period in 2025 (65 cases compared with 26 cases). WNV circulation is currently most intense in the Attica NUTS 2 region (48 cases), with Anatoliki Attiki (East Attica, 34 cases) and Voreios Tomeas Athinon (North Athens, seven cases) most affected.

As at 5 August 2026, locally acquired human cases of WNV infection had been reported in 58 NUTS 3 regions across seven countries. This is higher than at the same point in 2025, when cases were reported in 40 NUTS 3 regions across six countries. However, the geographical spread observed so far in 2026 remains well below the final extent recorded in recent seasons: by the end of 2025, affected regions numbered 160 and in 2024 there were 218. The 2024 season remains the largest WNV season on record in terms of geographical spread.

This year, six regions reported locally acquired human cases of WNV infection for the first time ever: **France** in Pyrénées‐Orientales (FRJ15); **Germany** in Rhein‐Pfalz‐Kreis (DEB3I); **Italy** in Campobasso (ITF22) and Viterbo (ITI41); **North Macedonia** in Pelagoniski (MK005) and Vardarski (MK001). Similar to previous years, most cases were reported among males aged 65 years and above. Most cases were hospitalised (72%) and presented with neurological symptoms (58%). The proportion hospitalised was lower than the average reported during the previous decade (87%), while the proportion with neurological symptoms was also slightly lower than the historical 10‐year average (64%). The case fatality rate was approximately 5%, lower than the average reported during the previous decade (11%). However, this estimate should be interpreted with caution, as clinical outcomes may not yet be known for all reported cases and additional deaths may be recorded as the season progresses and data are consolidated.

Owing to delays in diagnosis and reporting, and because most WNV infections are asymptomatic or subclinical, the reported case numbers probably underestimate the true number of infections. Seasonal surveillance in humans primarily captures laboratory‐confirmed cases, which may further contribute to reporting delays.

**Findings from veterinary surveillance:**

From the veterinary perspective, 17 WNV outbreaks among equids and 74 outbreaks among birds have been reported in Europe in 2026. The earliest start date of an outbreak among equids and birds was on 30 March 2026 in France and 31 March 2026 in Italy, while the latest onset of an outbreak among equids and birds was, respectively, on 31 July 2026 in Netherlands and 28 July 2026 in Italy. Outbreaks among equids were reported by **France** (five outbreaks), **Greece** (five outbreaks), **Italy** (five outbreaks), **the Netherlands** (one outbreak) and **Spain** (one outbreak). Outbreaks among birds were reported by **Italy** (64 outbreaks), **France** (five outbreaks), **Spain** (three outbreaks), **Austria** (one outbreak) and **Belgium** (one outbreak).

No information was available on the equid species involved in the outbreaks reported in the Animal Disease Information System (ADIS). The bird species most frequently associated with the reported outbreaks were the common magpie (23) and the hooded crow (20), followed by the carrion crow (5), the common kestrel (5), the Eurasian blackbird (5), the yellow‐legged gull (3), Adalbert's eagle (2), the common wood‐pigeon (2), and the little owl (2). Single outbreaks were associated with the common loon, the common raven, the European turtle‐dove, the golden eagle, the grey heron, the house sparrow and the northern goshawk.

The monthly number of WNV outbreaks in equids reported during the first part of 2026 was comparable to the corresponding 10‐year monthly average (2016–2025). However, the number of equid outbreaks in July 2026 remained below the levels observed in July 2018, 2024 and 2025, years characterised by particularly high WNV intensity. In contrast, the number of WNV outbreaks in birds slightly exceeded the corresponding four‐year monthly average (2022–2025) in April and May, and was substantially higher in June. This trend reversed in July 2026, when the number of reported outbreaks in birds fell below the four‐year average, although it remained higher than in July 2025. However, it should be noted that reporting delays may affect the July numbers, as some outbreaks occurring during that month may be notified in August.

As at 5 August 2026, outbreaks in birds and/or equids have been reported in 43 NUTS 3 regions across seven countries. This compares with 55 regions (10 countries) during the same period in 2025 and 42 regions (eight countries) in 2024. All seven countries reported WNV outbreaks in birds and/or equids in 2025 and in prior years, reflecting endemic WNV activity in these territories. However, as at 5 August, outbreaks in birds and/or equids were reported to ADIS for the first time in the following six regions: by France in Hauts‐de‐Seine (FR105) and Seine‐et‐Marne (FR102); by **Belgium** in Arr. Namur (BE352), by **Greece** in Drama (EL514), by **Italy** in Genova (ITC33) and by **the Netherlands** in Delf en Westland (NL362).

**Patterns across human and veterinary surveillance:**

Four countries – **France**, **Greece**, **Italy**, and **Spain** – reported both human WNV infections and outbreaks in equids and/or birds. As at 5 August, **Italy** accounted for most of the reported human cases (56.7%) and animal outbreaks (75.8%). **Greece** reported the second‐largest share of human cases (26.5%) but only five equid outbreaks and no bird outbreaks, representing 5.5% of all reported animal outbreaks.

Differences in the patterns observed across European countries may reflect a combination of ecological, climatic and surveillance‐related factors. Favourable climatic conditions and the presence of ecological hotspots, such as wetlands and agricultural areas, may support mosquito vector populations and influence the distribution and behaviour of animal hosts, thereby facilitating WNV circulation. At the same time, differences in WNV surveillance systems across Europe may affect detection and reporting rates.

The first indication of WNV activity may arise from either human or animal surveillance, depending on local epidemiology, detection capacity and surveillance system sensitivity. Therefore, the absence of reports from one sector should not be interpreted as evidence that WNV is not circulating. In **France** (Pyrénées‐Orientales, FRJ15), **Greece** (Drama, EL514), and **Italy** (Genova, ITC33), human cases were reported before, or in the absence of, notified outbreaks in birds or equids. Conversely, in **Belgium** (Arr. Namur, BE352), **France** (Hauts‐de‐Seine, FR105 and Seine‐et‐Marne, FR102) and **the Netherlands** (Delft en Westland, NL362), animal detections preceded human cases. **Belgium** is a relevant example, as WNV was first reported in the country in 2025 through avian surveillance, with no previous detections in humans and animals. In 2026, it has again been identified in birds in a previously unaffected region, highlighting the role of avian surveillance in detecting local virus circulation and geographical spread.

Active mosquito surveillance is also important for the early detection of WNV circulation. However, results on WNV detection in mosquitoes are not included in this report because they are not legally required to be reported at European level, and the available information is therefore scattered and often project‐based.

**Seasonal outlook:**

Given the favourable weather conditions for WNV transmission in Europe, ECDC and EFSA expect further human cases and outbreaks in equids and birds to be reported in the coming weeks. In previous years, transmission has typically peaked in August and September.

ECDC and EFSA continue to closely monitor the situation in Europe.

